# Stopping Murder by Medicine: Introducing the Model Law on Medicine Crime

**DOI:** 10.4269/ajtmh.15-0154

**Published:** 2015-06-03

**Authors:** Amir Attaran

**Affiliations:** Faculties of Law and Medicine, Institute of Population Health, University of Ottawa, Ottawa, Ontario, Canada

## Abstract

The iatrogenic pandemic of untreated illness related to falsified and substandard medicines is intolerable, but has a logical explanation: in many countries, inadequate laws make it barely illegal to manufacture or distribute poor-quality medicines. The law hardly punishes those who intentionally or recklessly deal in falsified or substandard medicine, when clearly it should criminalize these perpetrators in proportion to the grievous—even fatal—injury they inflict on public health. To solve this omission, this article presents a new *Model Law on Medicine Crime*, which countries may freely use as a template for strengthening their national laws. The *Model Law* includes criminal prohibitions against manufacturing, trafficking, or selling poor-quality medicines; principles for appropriately punishing offenders; special provisions for Internet-based medicine crimes; tools for encouraging whistle-blowers to cooperate with law enforcement; incentives for developing governments to strengthen their drug regulatory capacity; and important exceptions to prevent the law being abused, such as to prevent the prosecution of legitimate medical researchers or to prevent good-quality generic medicines being seized while in transit. The Model Law is discussed and explained and is offered free of charge under a Creative Commons license to any governments wanting to implement it.

## Introduction

The problem of criminally substandard and falsified medicines is the dark side of globalization and health. Rising wealth and open borders mean that more people than ever before can access medicines—an overwhelmingly good thing for public health—but wealth and open borders also attract organized criminals, who traffic falsified, substandard, or counterfeit medicines globally, leaving fraud, injury, and even death in their wake. As other articles in this special edition of the *American Journal of Tropical Medicine and Hygiene* relate, so far the salutary and criminal dimensions of the medicine trade appear inseparable—in some cases with deadly results.

But the relationship does not need to be Janus-faced as this. Today, much of the medicine crime problem exists because the laws themselves are unbalanced: the free trade laws that cause medicines to be globally traded are not matched by criminal laws to prosecute those who knowingly or negligently deal in bad quality medicines. To use a metaphor, free trade laws have opened numerous doors, but without adequate criminal laws, no guards are keeping watch at those doors. Insecurity and danger are the natural consequences of this halting, incomplete version of globalization.

Considering that medicine crime can kill, today’s laws are unreasonably weak. For example, in France, possessing a falsified medicine without legitimate reason attracts at most a €75,000 fine and 3 years’ imprisonment (and businesses, not able to be imprisoned, will only face fines).^1^ If that sounds too little, it far exceeds Norway, where imprisonment is at most 4 months.^2^ Worst of all, in the Netherlands, making a substandard medicine is not criminal at all, unless the offender does it twice in 2 years—the first violation is treated as just an administrative problem—and even then the imprisonment is at most 6 months.^3^ When supposedly advanced European countries have laws as feeble as this, naturally the poor countries, with less to invest in law enforcement systems, tend to do little or nothing at all.

Therefore, those who want to fight medicine crime because it endangers public health cannot realistically limit themselves to the scientific or medical dimensions, but must advocate for law reform that meaningfully stigmatizes the crime of trading in bad quality medicines and punishes the perpetrators. Ideally, countries should have laws that suppress both negligently substandard and deliberately falsified medicines without interfering with the legitimate trade in either branded or generic medicines. The public health community, the law enforcement community, and the pharmaceutical industry should all be able to agree on this goal.

However, and to complicate matters, some of the same European countries that hardly punish medicine crime at home are very aggressive when they perceive it abroad, as a backhanded way of blocking foreigners buying generic medicines. In a series of notorious incidents, customs officials at European airports seized dozens of shipments of legitimate, Indian-made generics, including antibiotics and antiretrovirals for acquired immunodeficiency syndrome (AIDS), while en route to Latin America and Africa; the cynical excuse was that these generics violated European patent or trademark laws for a brief moment while changing airplanes on European soil.^4^ Such ill-considered European actions, done for commercial reasons rather than to defend public health, generated understandable fury that set back the trust and political will needed to fight true medicine crimes—those caused by perilously bad quality medicines, rather than intellectual property violations—by several years.

This issue of the *American Journal of Tropical Medicine and Hygiene* presents an alternative to the lamentable status quo in which true medicine crimes are largely unaddressed, or are wrongly conflated with intellectual property issues. The web supplement of this article (hosted at the Social Science Research Network) contains a draft *Model Law on Medicine Crime*, which is the first effort of its kind to provide countries with a template that they can use to update their laws—a template that focuses strictly on medicine quality and health protection, while totally avoiding the controversial intellectual property issues. This article succinctly reviews its approach and features.

## The Model Law’s Basic Approach: Backward Compatibility

Any model law requires tailoring to fit local circumstances. Each country has its own unique legal and medical traditions, prevailing social, economic, and cultural conditions, and constitutional principles that no model law can fully address. For example, legal approaches that are natural in a common law system may appear odd in a civil law system or Islamic law system. Therefore, similar to one-size-fits-all clothing, the *Model Law on Medicine Crime* should be thought of as a starting point only: a proposal or first draft, which legislators may freely copy, edit, and tailor into a law that fits their national context.

To make the legislators’ job of tailoring easier, the design philosophy of the *Model Law* emphasizes *backward compatibility* with a country’s preexisting existing laws. For example, the *Model Law* does not lay down a system for registering or approving new medicines, because most countries already have such a system in their drug regulatory laws, and it is better for the *Model Law* to mesh with these existing laws than to clash with them by imposing something new and incompatible. Similarly, the *Model Law* does not contain a legal definition of the word “medicine” (or “drug”) because it is assumed that all countries already define that foundational word somewhere in their laws, and forcing them to adopt a new, probably different definition would have unpredictable or unintended consequences on existing laws.

Simply put, the philosophy of backward compatibility means that the *Model Law* is flexible enough not to depend on a specific regulatory system, a particular legal definition of a “medicine,” or many assumed parameters at all. This makes the *Model Law* much easier to implement than previous legal templates, such as those proposed by the United Nations Office of Drugs and Crime (UNODC) or the Council of Europe (CoE), which contain many top-down edicts that countries must follow. For example, the definition of a “medicinal product” in the CoE’s *MEDICRIME Convention* is the fulcrum on which all the criminal offences in that document depend; countries must adopt it exactly, without reservation, or the *MEDICRIME Convention* cannot work as intended.^5^ The *Model Law* rejects such straitjacketed thinking, and in being backward compatible, it follows the approach of the World Health Organization’s (WHO) legal advisors, who recommended using preexisting legal features “as defined in national legislation.”^6^ Thus the *Model Law* can fit easily into almost any country’s legal system.

## The Legal Measures

The actual legal provisions of the *Model Law* are divided into nine thematic parts. In brief, these include: some definitions of key terms; some prohibited acts that count as crimes; some penalties and principles for punishing those who engage in the crimes; some special rules for unique situations such as Internet pharmacies, medicines used in humanitarian emergencies, or medicine crimes that injure victims or evolve antimicrobial resistance; and some jurisdictional limits on all of the above. Together with some administrative sections, for example to excuse scientific researchers from prosecution or to reward whistle-blowers who report crimes, these elements make up the *Model Law*.

Part I of the *Model Law* contains key legal definitions. The most important are the definitions of “substandard” and “falsified” medicines, which are based on the work of a consensus group of lawyers, health professionals, drug regulators, and diplomats.^7^ Basically, substandard medicines are merely faulty, but falsified medicines are faulty due to intentionally wrongful conduct (another way of saying this is that falsified medicines are intentionally substandard medicines). The distinction between a substandard and a falsified medicine is therefore based purely on criminal intent, such as fraud or gross negligence, and does not depend on the chemical makeup of the medicine (Box 1

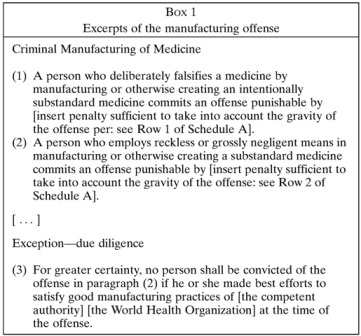

). For example, a medicine that contains degraded ingredients could be substandard (and not criminal) if it was caused by a purely unintentional handling error in transportation or storage, or could be falsified (and highly criminal) if someone intentionally took an old and expired medicine but relabeled it to appear new.

Although it may sound odd to persons who are not lawyers to base distinctions on intent rather than tangible physical properties, actually this is a bedrock principle of criminal justice. Consider, for example, the distinctions between striking and killing someone while driving a car at the speed limit (accidental homicide), striking and killing them while speeding (negligent homicide), or striking and killing them intentionally (murder). The physical result (death) is the same, but the intent is immensely different, and obviously justice cannot overlook the gradations.

Similarly, the *Model Law* contains prohibitions and penalties that are calibrated in accordance with intent, so as to distinguish acts that are merely accidental, criminally negligent, or criminally intentional. The *Model Law’s* definitions of “substandard” and “falsified” furnish the cornerstones of that distinction. The definitions are worded broadly enough to cover any sort of medicine: for example, allopathic medicines, traditional medicines, experimental medicines, compounded medicines, and even ingredients of medicines. Part I also contains definitions of “unregistered” medicines, which are those not approved by the local drug regulatory authority, and “counterfeit” medicines, which are those that violate trademark laws (although in this definition counterfeit medicines affect only intellectual property and are not the subject of the *Model Law*).

Crucially, all these definitions in Part I follow the philosophy of backward compatibility. The *Model Law* imposes no normative judgment about which medicines are falsified, substandard, unregistered, counterfeit, and so forth, but rather bases that nomenclature on a country’s existing rules: for example, a substandard medicine is one that fails to meet the required specifications of the country’s medicine regulatory authority. Thus, even if countries use different nomenclature than the *Model Law*, their existing rules are usually enough to make the nomenclature function, making it easy to implement the *Model Law* into real law. For example, at least four of the five permanent members of the UN Security Council (the United States,^8^ United Kingdom,^9^ China,^10^ and France^11^), plus the bulk medicine-exporting country, India,^12^ already criminalize wrongful acts involving medicine that are intentional or reckless—which is tantamount to criminalizing falsified medicines and recklessly substandard medicines in the *Model Law’s* nomenclature.

Part III of the *Model Law* is its criminal law core, containing the offences. These include the obvious crimes of intentionally manufacturing, advertising, selling, or otherwise trafficking falsified medicines, as well as the packaging of medicines or paraphernalia thereof. There are also similar, lesser offences involving substandard medicines, but these only come into play when a legitimate pharmaceutical trader behaves recklessly or negligently. Importantly, mere accidents are *not* criminalized, and to underscore this, it is clearly stated that anyone who is well-meaning and make best efforts to comply with good manufacturing practices commits no crime (Box 1). Part III also contains certain allowances that for practical reasons are especially important to developing countries, such as to stipulate that unregistered medicines should not be seized while *en route* to a lawful destination (Box 2

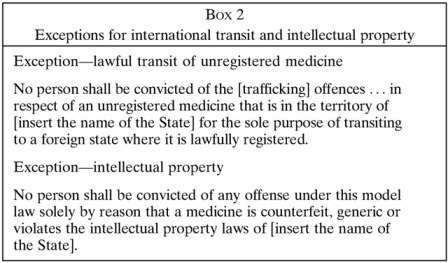

) and to allow the use of unregistered medicines (which normally are illegal) when there is an urgent need in disaster relief operations because there is no alternative. All these offences can apply both to natural persons (humans) and legal persons (corporations), whether they are the primary architect of the crime or supporting players in an organized criminal conspiracy, for example, by aiding a crime.

Part IV of the *Model Law* deals with the illicit Internet trade in medicines by Internet “pharmacies,” many of which are anonymous enterprises not run by real pharmacists at all. The *Model Law* makes it a crime to operate an Internet pharmacy in this dishonest fashion and requires compliance with all local pharmacy laws—or possibly double compliance, if the pharmacy and the medicine buyer are located in two different places (Box 3

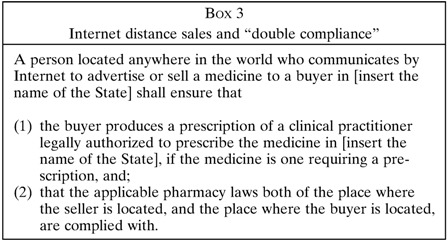

). Double compliance ensures safety throughout the supply chain, both at the point of sale and the point the patient consumes medicine. Part IV also criminalizes third parties who knowingly aid the illegal operations of an Internet pharmacy: for example, an Internet domain name registrar who refuses to suspend the web address of an illegal Internet pharmacy.^13^ Because the Internet makes it feasible to conduct crimes from certain “haven” countries that refuse to enforce the law, Part IV contains a new innovation not found in any law currently: courts may order the seizure of credit card payments and other financial transfers at the source, thus starving criminal Internet pharmacies and domain name registrars of revenue (Box 4

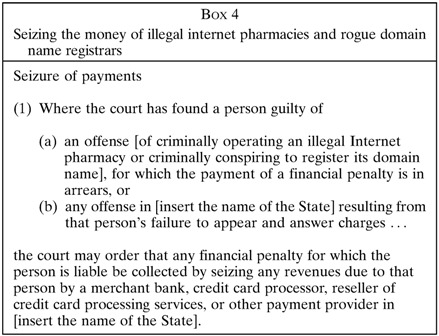

). That step alone can shut down a criminal operation and protect public health, even if arresting perpetrators hiding in a haven remains elusive.

Parts II and IV of the *Model Law* are its jurisdictional provisions. These are specially conceived to cast a long reach, commensurate with medicine crime itself. For example, in a recent criminal investigation crossing many borders, an Internet pharmacy in Canada advertised falsified bevacizumab (Avastin^®^) that was sourced in Turkey and distributed to patients in the United States through accomplices in at least four other countries.^14^,^15^ Faced with this sort of transnational crime, law enforcers need jurisdiction that can cross borders too, and so the *Model Law* lets them investigate, prosecute, or extradite criminals anywhere in the world, provided that either the criminals or their victims are connected with the law enforcing country through citizenship or place of residence. Although extraterritorial jurisdiction of this kind is unusual, it is being used to fight other serious or life-threatening crimes such as child pornography, human trafficking, war crimes, and perhaps most closely related, the making of fake (counterfeit) money.^16^

Parts V, VII and Schedule 1 of the *Model Law* set out principles for punishing crimes. As already mentioned, because deliberately perpetrating a crime is more deplorable than doing so through negligence, Schedule 1 sets out a penal hierarchy in which offences involving intentionally falsified medicines generally are punished more severely than offences involving negligently substandard ones. However, the *Model Law* leaves countries totally free to choose the manner and degree of punishment that is appropriate within this hierarchy, emphasizing imprisonment or fines as they think appropriate.

Part V adds some public health context to the penal hierarchy (Box 5

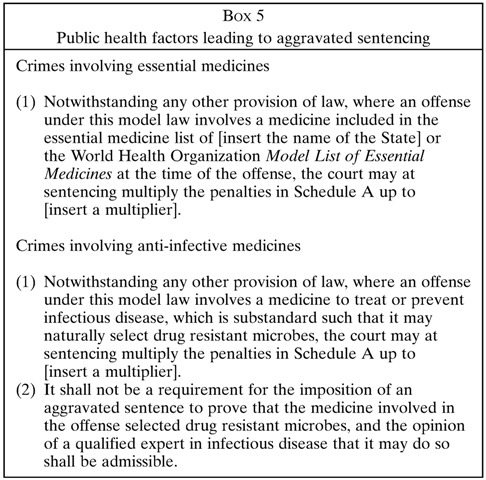

). It sets out a number of factors that can justify heavier or lighter punishment (or what lawyers call aggravation and mitigation of sentencing). Some of these factors are obvious and routine for the criminal law: for example, an unrepentant repeat offender might be punished more severely than a first-time offender who admits guilt and cooperates with law enforcers. The innovation of Part V is to expand on the usual criminal law factors, by adding public health considerations into the punishment decision. For example, when a criminal targets the medicines on WHO’s *Model List of Essential Medicines*, being predominantly inexpensive generic drugs needed by billions of patients, that person deserves to be punished more severely, commensurate with the scale of the attack on public health. Similarly, criminals who falsify poor-quality anti-infective medicines, many of which are chemically subtherapeutic and can select and evolve drug-resistant pathogens, deserve severer punishment.^17^ Aggravated sentencing also applies to medicine crimes that harm or kill patients, especially if the intent was for the crimes to be widespread or systematic in nature, which makes them analogous to a crime against humanity. Simply put, if a criminal act especially endangers public health, the *Model Law* allows for a higher punishment accordingly.

Part VI contains rules to guide the administration of justice. These include some pragmatic exemptions to ensure that the *Model Law* is not abused is ways that are overzealous, disproportionate, or unjust. Most important are the exemptions that forbid prosecutions of persons who commit technical violations of the law, but for understandable reasons, because without pragmatic exemptions the *Model Law* could be abused to prosecute scientists using placebos in a clinical trial (because placebos are intentionally mislabeled to deceive, which technically makes them falsified), or even patients who stash a few pills of “medicine A” in a bottle labeled “medicine B” for their personal convenience (and who among us has never done that when packing for a business trip or holiday). Importantly, Part VI also forbids prosecutions where the only issue is that a medicine is generic or violates trademarks, and this is to ensure that the *Model Law* remains a tool for protecting public health and is not turned into a cudgel for enforcing intellectual property (Box 2).

Part VI also contains innovative provisions to give a financial reward to whistle-blowers who report medicine crime. Although few countries today reward whistle-blowers, the tactic can be very effective: arguably the most sweeping medicine crime prosecution in history began with a whistle-blower and ended with a major pharmaceutical company (Ranbaxy, Gurgaon, India) pleading guilty to various criminal acts, including selling adulterated, falsified medicines based on fraudulent safety testing.^18^ The company paid fines and penalties of $500 million, which more than justified the reward paid to the whistle-blower.

Parts VII and VIII of the *Model Law* contain a number of additional punitive, restorative, or preventive measures against medicine crime. There are powers for law enforcement to confiscate the ill-gotten wealth of medicine criminals, to destroy stocks of illicit medicines, and to extradite accused criminals to face prosecution for crimes committed abroad. On the prevention side, countries are encouraged to designate only a few, selected ports to process medicine imports—a measure that can help turn those ports into centers of excellence for detecting medicine crime, and which has the convenient side effect that it automatically criminalizes any smuggling that bypasses those ports. Port controls of this kind have helped Nigeria reduce the number of illegal medicines on its market.^19^

Finally, Part IX contains transitional measures for the 30% of countries that, according to WHO, have no or very limited medicines regulation.^20^ Such countries have few, if any, registered medicines on the market, meaning that health care routinely depends on unregistered medicines. Obviously, it would be immoral and inhumane of the *Model Law* to criminalize doctors, pharmacists, and patients who have no option but to use unregistered medicines, and so Part IX makes allowances for this fact through a new mechanism called the regulatory recognition order. This mechanism allows the authorities to legalize unregistered medicines of known good quality for a temporary duration (up to 2 years). A similar sort of regulatory recognition order is available to legalize unregistered medicines immediately in cases of humanitarian emergency (e.g., an earthquake, typhoon, or war) so that airlifts of lifesaving medicine are not held up for legal reasons.

Regulatory recognition orders of these sorts not only prevent unjust prosecutions against health care and emergency relief workers but also offer a rudimentary form of medicine regulation for countries currently lacking it and can serve as a stepping-stone toward instituting a full-blown medicine regulatory authority. In that sense, the *Model Law* is not strictly about criminalizing bad quality medicines, but also building the regulatory capacity in countries so that good quality medicines eventually become the norm.

## The Path Ahead

For the *Model Law on Medicine Crime* to have a public health impact, it has to be emulated by legislators passing real laws. For that reason, the *Model Law* in the web supplement is published under a Creative Commons Attribution-ShareAlike license, so that anyone may freely copy, modify, and republish their own version, provided that they cite the original author and extend these same courtesies to others. In time, improvements can be made and shared. This approach to creating public goods has been very successful in other creative fields—for example, open-source software—and is worth trying for public health law.

I believe that for the medicine crime problem to be solved, law reform is an absolutely necessary condition (although not a sufficient condition, especially if law enforcement is lacking). But law reform is extremely unlikely without the advocacy and lobbying of the public health community, for who else can do it?

There is precedent. Probably the greatest public health success in living memory—tobacco control—came about because the public health community pushed for law reform. After research, the public health community lobbied and demanded laws that limited tobacco advertising, forbade public smoking, and redesigned tobacco packaging. Then the public health community watched over governments to ensure those hard-won laws were enforced. These successes were achieved despite the extremely well funded counter-lobbying of the tobacco industry.

Now, in comparison, reforming the criminal law to address unsafe, bad quality medicines should be much easier. Medicine criminals have little lobbying power, and the law-abiding pharmaceutical industry (whether branded or generic) also want to see the criminals vanquished and could even be allies. Currently, it is the public health community’s own lack of drive, and not outside opposition, which is rate-limiting.

However, for the public health community to achieve a meaningful success, it must avoid the mistakes that have thwarted others. Within the last decade, the UNODC and the CoE also developed model legislation, but did so with such secrecy and conflict of interest that they poisoned the outcome: both settled on legal texts that broadened the enforcement of pharmaceutical intellectual property rights, probably because they invited a single French pharmaceutical company (Sanofi, Paris, France) and its nongovernmental organization (NGO) offshoot (called the International Institute of Research Against Counterfeit Medicines, or IRACM) into their drafting processes, which they conducted behind closed doors and with scant representation from developing country officials or health professionals such as doctors, nurses, or pharmacists.^21^–^23^ Without expert inputs like these, the UNODC and CoE legal texts made foolish mistakes, such as to permit seizing generic medicines while in transit and to criminalize scientists and health care workers who use using placebos in clinical research.^22^,^24^ Outcomes like these understandably inflame developing countries, and even led Brazil, China, India, Russia, and South Africa to file formal diplomatic protests.^25^ Because the public health community can work in a more transparent, inclusive fashion, it can have a better outcome.

Medicine crime causes iatrogenic injury to patients, which makes it intolerable. I suggest that the public health community should fight it through a sustained campaign by its professional associations: for example, national associations of public health or medicine, and their global affiliates such as the World Federation of Public Health Associations, the International Pharmacists Federation, the International Council of Nurses, and the World Medical Association. These organizations have already been outspoken against medicine crime, although none of them have yet lobbied for law reform.^7^,^26^ Yet, history teaches that law reform is the obvious next step when translating research into action for health protection, just it was for tobacco. This *Model Law* is offered as a guide, in the hope that a similar victory can be achieved for the sake of all patients who depend on medicines for their well-being.
